# Mediating Effect of Social Support on the Association between Functional Disability and Psychological Distress in Older Adults in Rural China: Does Age Make a Difference?

**DOI:** 10.1371/journal.pone.0100945

**Published:** 2014-06-25

**Authors:** Danjun Feng, Linqin Ji, Lingzhong Xu

**Affiliations:** 1 School of Nursing, Shandong University, Jinan, China; 2 School of Psychology, Shandong Normal University, Jinan, China; 3 Department of Health Services Management and Maternal & Child Healthcare, Shandong University, Jinan, China; Institute of Psychiatry, United Kingdom

## Abstract

This study aimed to determine the prevalence of psychological distress among elderly people in rural China. Moreover, the mediating effect of social support on the association between functional disability and psychological distress and whether this effect varies with age would be examined. A total of 741 elderly people aged 60–89 years from a rural area of Shandong Province, China participated in a cross-sectional survey. Their psychological distress, perceived social support, enacted social support, and functional disability were assessed through questionnaires. A total of 217 (29.3%) rural elderly people had psychological distress. The functional disability of people ≥75 years old had smaller total effects (0.18) on their psychological distress than in people <75 years old (0.30). Moreover, most of the effects of functional disability on psychological distress among the people ≥75 years old were indirect (0.12; 66.67% of total effects) through the mediating effect of social support especially perceived support, while the direct effect of functional disability was insignificant. In contrast, most of the effects of functional disability on psychological distress among the people <75 years old were direct (0.29; 96.67% of total effects), while the mediating effect of social support was insignificant. In conclusion, the total effect of functional disability, especially the direct effect, on psychological distress decreases sharply with age. The mediating effect of social support on the association between functional disability and psychological distress varies with age and is only found in people ≥75 years.

## Introduction

The results of the latest National Census in 2010 indicate that China has the largest elderly population in the world, with 117.6 million people aged 60 and over, representing 13.26% of the total population of China and more than 20% of the world's total elderly population. In addition, China is projected to age much faster than Western countries in the near future: about 25% of the total population will be aged 60 and over by 2030 [1].

China is a typical agricultural country, with 60% of the elderly population living in rural areas. Traditionally, there have been large gaps between urban and rural areas in China with respect to economic and social development. China has had one of the highest urban–rural income ratios worldwide until recently [2]. Elderly people residing in rural areas have a much lower education level than their urban counterparts. Moreover, less-educated elderly Chinese people have higher levels of distress than their better-educated counterparts [3]. Thus, rural-dwelling elderly people in China usually have lower socioeconomic status than urban elderly people, and these conditions increase the rates of mental disorders [4].

Psychological distress is a nonspecific negative psychological state that includes combined feelings of anxiety and depression. In addition to being significantly associated with mental disorders [5], psychological distress also affects a greater percentage of the population than mental disorders. Whilst several studies have investigated the prevalence of pure anxiety or depression in rural older Chinese adults [6], [7], no study has assessed their prevalence of psychological distress. The present study aims to fill this knowledge gap.

Elderly people in rural China have a higher prevalence of functional disability than their urban counterparts [8]. Functional disability is among the most significant risk factors for psychological distress [9], [10]. Nevertheless, The effects of functional disability on one's psychological distress may vary as a function of the time or age when functional disability developed. The time which does not conform to socially based expectations of normative timing may be viewed as being “early” or “late” from a life course perspective [11]. These norms are likely to exacerbate the stress induced by functional disability by increasing their undesirability for individuals at younger stages of late life [12]. On the contrary, a higher prevalence of functional disability among age peers may result in a greater desensitization to these problems among the older elderly group [13].Therefore, the effects of functional disability on distress may decrease with aging. However, current empirical evidence is inconsistent. One study supported this viewpoint and reported that increasing age reduces the strength of the association between functional disability and depression [14]. Another study reported that functional disability has a stronger effect on distress in older age in some people [15]. There was also one study which reported that age moderates the relationship between functional disability and depression only among elderly with higher perceived social support [16]. Therefore, the role of social support should be taken into account when studying the moderating effect of age on the relationship between functional disability and psychological distress.

The moderating or buffering effect of social support on the relationship between stressors including functional disability and mental health is well studied and supported [17], [18]. However, its mediating effect is less studied. Results regarding the mediating effect of social support on the relationship between stressors and mental health are inconsistent. Ensel and Lin propose 2 models of this mediating effect [19]. One is called the “counteractive model,” which assumes that stressors mobilize social support that suppresses the effects of stressors on distress. The other is called the “deterioration model,” which postulates that stressors exhaust social support and thus support cannot continuously suppress the effects of stressors on distress. One study tested these 2 competing models in a sample of African-American college students, and supported the deterioration model [20]. In contrast, another study partly supported the counteractive model [21]. They found that stress leads to increased social support; however, only one type of social support (i.e., practical help) significantly reduced distress among widows. Yange reports that functional disability, as a specific stressor, leads to both higher instrumental social support and lower perceived social support [10]. However, only perceived social support was found to significantly affect a person's depression; this suggests that different types of social support have different mediating effects on the relationship between functional disability and psychological distress. Therefore, the present study also examined the mediating effects of 2 usual types of social support—perceived support and enacted support—on the association between functional disability and psychological distress, and whether these mediating effects vary by age among rural elderly Chinese.

## Methods

### Ethics Statement

Written informed consent was provided by all the participants, and the study was approved by the Ethics Committee of School of Public Health in Shandong University, P. R. China.

### Participants

The participants were recruited from 3 counties in Dongying City, Shandong Province in Eastern China using 2-stage random sampling. In the first stage, 3 towns were randomly selected from each county for a total of 9 towns. By employing the same procedure, 3 villages from each town were sampled in the second stage. Finally, a total of 27 villages were sampled. All residents aged 60 and above in the sample villages were approached to be included in the study. As such subjects have great difficulties finishing the questionnaires independently because of their lower education level (only 17.1% have finished high school education), the investigators conducted face–to-face interviews using a structured questionnaire. A total of 741 valid questionnaires were obtained, representing an 87.18% response rate. The sociodemographic characteristics of the sample are presented in Table 1.

**Table 1:** Prevalence of psychological distress according to sociodemographic variables.

Groups	n(%)	Psychological distress(≥16)(n,%)	*p*
Age group			
Younger elderly (<75)	609(82.2)	176(28.9)	
Older elderly (≥75)	132(17.8)	41(31.1)	>0.05
Gender			
Men	396(53.4)	91(23.0)	
Women	345(46.6)	126(36.5)	<0.001
Education level			
No school education	393(53.0)	140(35.6)	
Primary school	221(29.8)	50(22.6)	
High school and college	127(17.1)	27(21.3)	<0.001
Marital status			
Widowed/single	145(19.6)	54(37.2)	
Married	596(80.4)	163(27.3)	<0.05
Total	741(100.0)	217(29.3)	

### Measures

The Kessler 10 (K10) was used to assess nonspecific psychological distress. This instrument has previously exhibited excellent psychometric properties [5], and the Chinese version has also been proven to have good reliability and validity [22], [23]. An example item is, “Have you often felt worn out without good reason in the past 30 days?” Responses are recorded on a 5-point scale ranging from 1 (never) to 5 (all the time); the total score is calculated by summing the responses. According to the Victorian Population Health Survey [24], scores >16 indicate moderate to serious psychological distress. The Cronbach's alpha of this instrument is 0.93.

Functional disability was assessed on the basis of 5 items reflecting difficulties in doing housework, dressing, physical pain, concentrating, and identifying acquaintances. An example item is, “Overall in the last 30 days, how much difficulty did you have with dressing yourself?” Responses were recorded on a 5-point scale ranging from 1 (none) to 5 (very much). The Cronbach's alpha of this instrument is 0.81.

Perceived social support was measured by 3 items inquiring about the participants' feelings about their relationships with friends, co-workers, and neighbors respectively. An example item is, “How do you feel about your relationships with your friends?” Responses were recorded on a 4-point scale ranging from 1 (never care for each other) to 4 (often care for each other). The Cronbach's alpha of this instrument is 0.74.

Enacted social support (i.e., support received from others) was measured by 4 items asking about the social support the participants have received. An example item is, “Where do you get financial assistance in emergencies?” Responses for these multiple-choice items included none, spouse, other family members, friends, relatives, workmates, employer, official or semi-official agency, non-government organization, and others. These items were scored on the basis of the number of chosen sources of social support. The Cronbach's alpha of this instrument is 0.79.

### Statistical analyses

Descriptive analyses were conducted to determine sociodemographic differences in the prevalence of psychological distress among elderly people in rural China. Then, Pearson product−moment correlations among functional disability, perceived social support, enacted social support, and psychological distress were calculated. All data were analyzed by SPSS version 18.0 (IBM Corporation, Armonk, NY, USA).

We also employed a structural equation modeling (SEM) approach with the bias-corrected bootstrap method (2,000 replicates) using AMOS version 18.0 (IBM Corporation, Armonk, NY, USA) to determine the influence of functional disability on psychological distress with social support as a mediator. SEM estimates both the direct and indirect effects a variable has on the outcome variables while simultaneously minimizing the effects of measurement error [25]. AMOS generates several indices to determine if the hypothesized model fits the observed data. The original method for assessing model fit is the *χ* ^2^ statistic. However, *χ* ^2^ is sensitive to sample size. Therefore, a normed *χ* ^2^chi-square (*χ* ^2^/*df*) was used to assess model fit; a good fit is indicated when *χ* ^2^/*df*<3. Several other indices of model fit were also used in the analysis. the root mean square error of approximation(RMSEA)<0.08 is acceptable and <0.05 is excellent. Goodness of Fit Index(GFI) should be >0.90 in a good-fitting model. Values of Comparative Fit Index (CFI) and the Tucker Lewis Index (TLI)>0.90 are considered to indicate acceptable fit.

**Figure 1:**
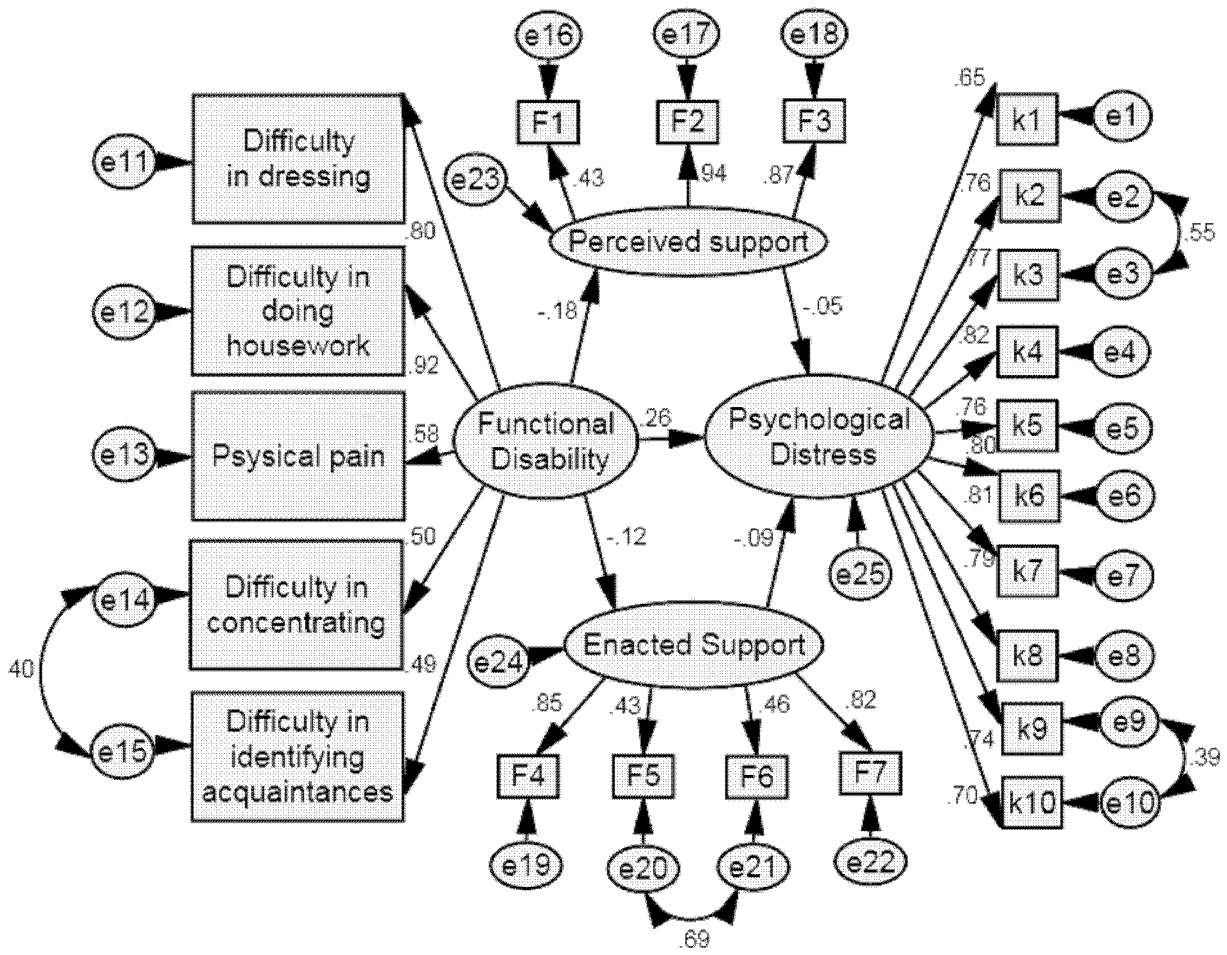
Results of SEM analysis among whole sample. All the coefficients are standardized. The path coefficient (−0.12) from functional disability to enacted support is significant at 0.01 level, that from enacted support (−0.09) to psychological distress are significant at 0.05 level, that from perceived support to psychological distress (−0.05) are not significant at 0.05 level and all other coefficients in the figure are significant at 0.001 level.

Finally, a multiple group analysis of SEM was conducted to test the invariance of the final model (see Figure 1) between participants divided into the younger and older elderly groups: <75 and ≥75 years, respectively. This analysis specifically imposes successive restrictions on the measurement weights, structural weights, structural covariance, structural residuals, and measurement residuals, forcing them to remain identical between the 2 age groups.

## Results

The prevalence of psychological distress with respect to sociodemographic characteristics is shown in Table 1. A total of 29.3% of participants scored >16 on the K10, indicating moderate or serious psychological distress. Women (36.5%) reported higher psychological distress than men (23%; *χ* ^2^_(1)_ = 16.33, *p*<0.001). Participants without any school education reported the highest psychological distress (35.6%). Married elderly (27.3%) had a lower prevalence of psychological distress than widowed or single participants (37.2%; *χ* ^2^_(1)_ = 5.51, *p*<0.05). There was no significant association between age and the prevalence of psychological distress.

The correlations among functional disability, perceived social support, enacted social support, and psychological distress are presented in Table 2. Psychological distress was positively correlated with functional disability and inversely correlated with perceived social support and enacted social support.

**Table 2:** Matrix of variables (means, standard deviations, ranges and correlations).

	M	SD	Range	1	2	3	4
1.Funtional disability	7.40	3.18	5.00–25.00	1			
2.Perceived social support	10.21	2.13	3.00–12.00	−0.14**	1		
3. Enacted social support	10.40	3.62	2.00–18.00	−0.12**	0.31**	1	
4.Psychological distress	14.67	6.68	10.00–44.00	0.29**	−0.14**	−0.13**	1

*Note*. **p<0.01.

The SEM analysis of the effect of functional disability on psychological distress with social support as a mediator among whole sample indicated that the data failed to support the theoretical model (*χ* ^2^/*df* = 7.88, *p*<0.001, RMSEA = 0.10, GFI = 0.83, CFI = 0.85, TLI = 0.83). In an attempt to develop a better-fitting model, post hoc modifications were performed with reference to the modification index. Several pairs of error terms were specifically correlated. As a result, the final model yielded a good fit of the data (*χ* ^2^/df = 3.56, *p*<0.001, RMSEA = 0.06, GFI = 0.92, CFI = 0.94, TLI = 0.94). Functional disability had both, a significantly direct (0.26, *p*<0.001) and a minor but significant indirect (0.02, *p*<0.05) effect on psychological distress (Table 3 and Figure 1).

**Table 3:** Results for the direct and indirect effects of functional disability on psychological distress with social support as mediator.

Sample	n	Effects	Point estimate (%)	95% bias-corrected CI
Younger elderly	609	Direct effect	0.29(96.67)	(0.17, 040) ***
		Indirect effect	0.01(3.33)	(−0.00, 0.03)
		Total effect	0.30(100)	(0.18, 0.41) ***
Older elderly	132	Direct effect	0.06(33.33)	(−0.16, 0.27)
		Indirect effect	0.12(66.67)	(0.02, 0.27) *
		Total effect	0.18(100)	(0.00, 0.36) *
Whole sample	741	Direct effect	0.26(92.86)	(0.16, 0.37) ***
		Indirect effect	0.02(7.14)	(0.01, 0.05) *
		Total effect	0.28(100)	(0.18, 0.38) ***

*Note*. CI, confidence interval. Biased-corrected bootstrap with 2000 replications.

*p<0.05,

**p<0.01,

***p<0.001.

**Figure 2:**
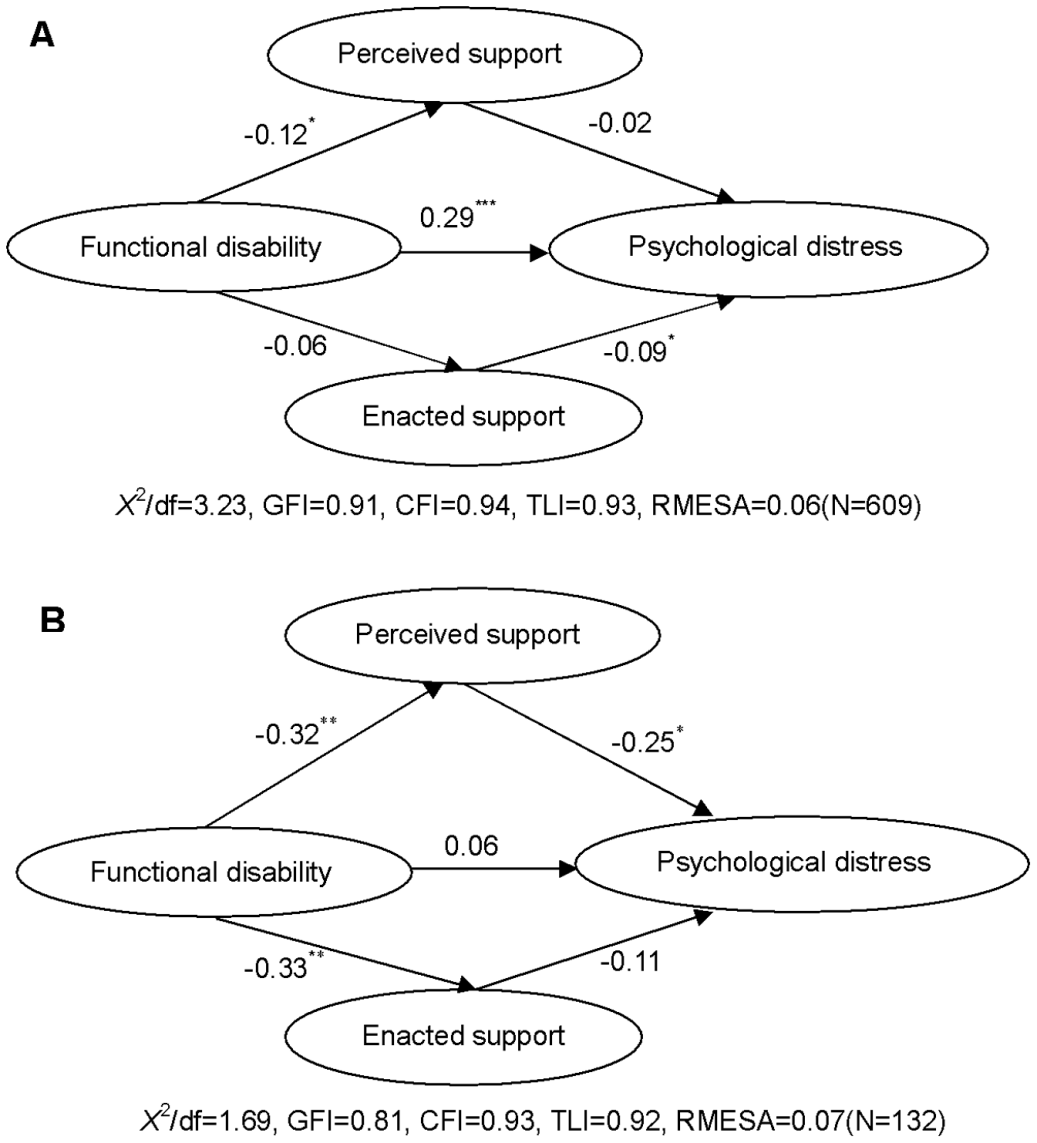
Results of SEM analysis among the people <75 years old (A) and the people ≥75 years old (B). All the coefficients in the figures are standardized. Observed indicators for the latent factors are not shown. *p<0.05, **p<0.01,*** *p*<0.001.

The multiple group analysis of SEM demonstrated that differences in goodness-of-fit statistics existed among the models with no restrictions, restricted measurement weights, restricted structural weights, and restricted structural covariance (see Table 4). This indicates that the relationships among functional disability, perceived social support, enacted social support, and psychological distress differ between the younger and older elderly groups. The results of SEM analyses of the effect of functional disability on psychological distress with social support as a mediator by age group (see Figure 2 and Table 3) demonstrated that functional disability exerted different effects on psychological distress via different paths in both groups. In general, functional disability had smaller total effects (0.18) on psychological distress in the older elderly group than the younger elderly group (0.30). In particular, most of the effects of functional disability on psychological distress among the elderly were indirect (0.12; 66.67% of total effects) for functional disability had a significant negative effect on both enacted support (*β* = −0.33, *p*<0.01) and perceived support (*β* = −0.32, *p*<0.01), with the latter leading to a significant decrease in psychological distress (*β* = −0.25, *p*<0.05). In contrast, although functional disability did not have a significant effect on enacted support (*β* = −0.06, *p*>0.05) and a significantly negative effect (*β* = −0.12, *p*<0.05) on perceived support, the latter had no significant effect (*β* = −0.02, *p*>0.05) on psychological distress. Hence, most of the effects of functional disability on psychological distress among the younger elderly group are direct (0.29; 96.67% of total effects), with the mediating effect of social support being insignificant.

**Table 4:** Goodness-of-fit statistics for the multiple group analysis.

Goodness-of-fit statistics	x^2^(df)	P	△x^2^(df)	P	GFI	CFI	TLI	RMSEA
Model with no restrictions	985.61(400)	0			0.89	0.94	0.93	0.05
Model with restricted measurement weights	1024.08(418)	0	38.46(18)	<0.01	0.89	0.93	0.93	0.04
Model with restricted structural weights	1041.85(423)	0	17.77(5)	<0.01	0.89	0.93	0.93	0.04
Model with restricted structural covariance	1052.26(424)	0	10.42(1)	<0.001	0.88	0.93	0.93	0.05
Model with restricted structural residuals	1054.54(427)	0	2.28(3)	>0.05	0.88	0.93	0.93	0.05
Model with restricted measurement residuals	1199.71(453)	0	145.11(26)	<0.001	0.87	0.92	0.92	0.05

*Note*. GFI, goodness of fit index. CFI, comparative fit index. TLI, Tucker Lewis Index. RMSEA, root mean square error of approximation.

## Discussion

To our knowledge, this is the first study in China to report the prevalence of psychological distress among rural elderly using the K10. The rate of psychological distress (i.e., K10≥16) in the present study was 29.3%, which is equal to that reported in elderly Australian people (>65 years, including both rural and urban residents) [24].

Concordant with the literature [26], elderly women had a higher prevalence of psychological distress than elderly men. The elderly people with less education reported the highest psychological distress, which is also consistent with a previous report [3]. There was no difference in the prevalence of psychological distress among rural elderly people with respect to age, which is also consistent with a recent study in Japan [27].

Unsurprisingly, functional disability had a significantly positive effect on psychological distress in both age groups when the indirect effects mediated by social support were taken into account. However, if only the direct effect is considered, the influence of functional disability on psychological distress in the older elderly group became insignificant. Even taking into account the indirect effects mediated by social support, the psychological distress of the older elderly group was less influenced by functional disability than that in the younger elderly group. These results are concordant with a study in America that also found a decreasing relationship between functional disability and depression with increasing age [14].

The mediating effect of social support on the relationship between functional disability and psychological distress only existed in the older elderly group. The functional disability of the older elderly people led to significant decreases in both perceived support and enacted support; however, only perceived support exerted a significant protective effect on mental health. These results partly support the deterioration model. However, in the younger elderly group, functional disability only reduced perceived support, which did not significantly affect psychological distress. These results indicate that the mediating effect of social support on the relationship between functional disability and distress varies with age. Interestingly, the present results show increasing age is associated with greater damage to social support caused by functional disability. Moreover, in younger elderly people, enacted support appears to play a more important role in protecting mental health than perceived support. In contrast, in older elderly people, perceived support appears to play a more important role in protecting mental health than enacted support. Nevertheless, further studies are required to confirm these findings before drawing conclusions.

Four pairs of error terms were significantly correlated in the SEM analysis (see Figure 1). In general, correlations among error terms should be interpreted cautiously. However, this practice is acceptable when supported by a strong theoretical framework [28]. Both items K9 (“how often do you feel that nothing can interest you?”) and K10 (“how often do you feel bored?”) are related to depressive personality; therefore, the correlation of their error terms is theoretically reasonable.

The present study has some limitations. The first limitation is related to the study's methodology. Because of the study's cross-sectional design, the causal paths in the model are still based on hypothetical relationships. Hence, a longitudinal study is required to ascertain causal relationships among variables. Second, all participants in the present study were from Dongying City, Shandong Province. Therefore, the sample is not representative of other rural regions in China considering China's vast size. Thus, further research with participants from a wider range of regions is required to determine the generalizability of the results to elderly people from other rural areas in China. Furthermore, in the current study, the direct effect of functional disability on psychological distress may operate through some variables which have not been measured and future studies are needed to explore these variables.

In conclusion, the total effect—especially the direct effect—of functional disability on psychological distress decreases sharply with age. The mediating effect of social support on the association between functional disability and psychological distress varies with age and is only found in people aged ≥75 years.
